# Evaluation of an early implementation of the revised Opening Minds Through Art Program

**DOI:** 10.1093/geroni/igaa057.2963

**Published:** 2020-12-16

**Authors:** Christopher Kelly, Lyn Holley, Stephen Fogle

**Affiliations:** University of Nebraska Omaha, Omaha, Nebraska, United States

## Abstract

The established international program Opening Minds through Art (OMA) has been revised; this presentation reports evaluation of an early implementation with Gerontology university students and Alzheimer’s nursing home patients. Ten patients were paired with student volunteers meeting once a week for eight weeks to co-create original artwork in structured one-hour sessions. Before and after art creation each volunteer recorded personal feelings and their partner’s mood and satisfaction. Findings indicate the revised program is satisfying for patients and improves their mood. Families seeing the art expressed surprise and appreciation regarding patient creative capacity. Analysis of data indicates positive outcomes for student volunteers and Alzheimer’s patients. Student volunteer reflections link program participation with expanded knowledge, insight, and especially empathy for Alzheimer’s patients and their families. The current study contributes to robust support in the literature for efficacy of arts programming for student learning and the morale of Alzheimer’s patients and their families.

